# Increased ATP Release and Higher Impact of Adenosine A_2A_ Receptors on Corticostriatal Plasticity in a Rat Model of Presymptomatic Parkinson’s Disease

**DOI:** 10.1007/s12035-022-03162-1

**Published:** 2022-12-22

**Authors:** Francisco Q. Gonçalves, Filipe C. Matheus, Henrique B. Silva, Joana I. Real, Daniel Rial, Ricardo J. Rodrigues, Jean-Pierre Oses, António C. Silva, Nélio Gonçalves, Rui D. Prediger, Ângelo R. Tomé, Rodrigo A. Cunha

**Affiliations:** 1 CNC-Center for Neuroscience and Cell Biology, University of Coimbra, Coimbra, Portugal; 2 Department of Pharmacology, Center of Biological Sciences, Federal University of Santa Catarina (UFSC), Florianópolis, SC Brazil; 3 Department of Life Sciences, Faculty of Sciences and Technology, University of Coimbra, Coimbra, Portugal; 4 Faculty of Medicine, University of Coimbra, Coimbra, Portugal

**Keywords:** Adenosine, Ecto-5′-nucleotidase, A_2A_ receptors, Parkinson’s disease, ATP

## Abstract

Extracellular ATP can be a danger signal, but its role in striatal circuits afflicted in Parkinson’s disease (PD) is unclear and was now investigated. ATP was particularly released at high stimulation intensities from purified striatal nerve terminals of mice, which were endowed with different ATP-P2 receptors (P2R), although P2R antagonists did not alter corticostriatal transmission or plasticity. Instead, ATP was extracellularly catabolized into adenosine through CD73 to activate adenosine A_2A_ receptors (A_2A_R) modulating corticostriatal long-term potentiation (LTP) in mice. In the presymptomatic phase of a 6-hydroxydopamine rat model of PD, ATP release from striatal nerve terminals was increased and was responsible for a greater impact of CD73 and A_2A_R on corticostriatal LTP. These observations identify increased ATP release and ATP-derived formation of extracellular adenosine bolstering A_2A_R activation as a key pathway responsible for abnormal synaptic plasticity in circuits involved in the onset of PD motor symptoms. The translation of these findings to humans prompts extending the use of A_2A_R antagonists from only co-adjuvants of motor control in Parkinsonian patients to neuroprotective drugs delaying the onset of motor symptoms.

## Introduction

ATP is an extracellular signaling molecule, which can act in the brain as a neurotransmitter, co-transmitter, gliotransmitter, or synaptic neuromodulator, and has a prominent role as a danger signal in the brain [1]. ATP can directly engage the function of different P2 receptors (P2R), classified into ionotropic P2XR and metabotropic P2YR, both of which can control information processing in brain circuits as well as format the onset or extent of damage upon brain insults or diseases [1, 2]. Additionally, extracellular ATP can also indirectly signal through adenosine receptors upon conversion of extracellular ATP into adenosine, through the action of ecto-nucleotidases including ecto-5′-nucleotidase or CD73 [3]. Adenosine is a prototypical neuromodulator, mostly acting in the brain through inhibitory A_1_ receptors (A_1_R) and facilitatory A_2A_ receptors (A_2A_R) [4]. Whereas astrocytic ATP release seems to be mostly associated with the activation of A_1_R to control basal synaptic transmission [4], the synaptic release of ATP is most evident at higher frequencies [5, 6] that are associated with synaptic plasticity processes involved in learning and adaptive processes, precisely the conditions where the engagement of A_2A_R is most evident in different brain areas [7]. Indeed, previous studies have concluded on a preferential association of synaptic ATP release with the selective recruitment of A_2A_R [8–11], probably resulting from a physical association of A_2A_R and CD73 [8]. Furthermore, since synaptic dysfunction and loss are core processes at the onset of most neurodegenerative disorders [12], the antagonism of A_2A_R is now recognized as a robust neuroprotective strategy in different brain disorders [13].

There is robust evidence that A_2A_R antagonism attenuates behavioral and neurochemical features of Parkinson’s disease (PD) (reviewed, e.g., in [14, 15]), which has heralded the recent approval by the FDA of the first A_2A_R antagonist to manage PD [16]. Furthermore, we have previously shown that an enhanced ATP release and CD73-mediated adenosine formation sustain a persistent A_2A_R overactivation in a rat model of PD [9]. In parallel, tinkering with P2R also modifies neuropathological and behavioral features of PD (e.g. [17],), which questions if ATP mostly acts directly on P2R or indirectly on A_2A_R after its extracellular catabolism into adenosine, especially at the onset of PD. Furthermore, although A_2A_R control abnormal synaptic plasticity processes at corticostriatal synapses [18, 19], this has not been established in PD models [20], where it is unknown if A_2A_R overfunction is an early process contributing for motor dysfunction or a maladaptive process resulting from motor dysfunction.

This study was designed to explore two intertwined questions: first, we took advantage of different mouse knockout models, to characterize the pattern of ATP release in synapses and whether it modulated synaptic plasticity through P2 receptors or upon its extracellular catabolism into adenosine. We next exploited our previous rat model of presymptomatic to symptomatic PD [21, 22] to define if this synaptic ATP-driven A_2A_R purinergic modulation is associated with abnormal corticostriatal plasticity at the onset of motor symptoms in PD.

## Materials and Methods

### Animals

Male C57bl\6j mice (8–12 weeks old; total of 26) and male Wistar rats (8–12 weeks old; total of 32) were from Charles River (Barcelona, Spain). A_2A_R-knockout (KO; total of 5) and CD73-KO mice (total of 5), both in a C57bl\6 background, were generated and crossbred as previously described [8, 10]. Animals were maintained in groups of two to five per cage in a temperature-controlled room (22 ± 1 ◦C), with free access to food and water, and with a 12-h light/12-h dark cycle (lights on at 7:00 am). Animals were handled according to ARRIVE guidelines, as approved by CNC Ethical Committee for Animal Research (ORBEA-138/2016). In all experiments, the experimenters were unaware of the experimental group to which each animal belonged. This exploratory study was not pre-registered.

### Drugs

The goal of this study was to evaluate the role of endogenously produced ATP and adenosine on corticostriatal synaptic function, which implies the use of antagonists of purine receptors and we purposely avoided the use of agonists, which could engage receptor populations that may not be recruited by endogenously produced purines under our experimental conditions. Brilliant Blue-G (BBG, Cat# B0770, 2019) and α,β-methylene ADP (AOPCP, Cat# M3763, 2019) were from Sigma (St. Louis, USA) and PPADS (pyridoxalphosphate-6-azophenyl-2′,4′-disulfonic acid, Cat# 0625, 2019), SCH58261 (2-(2-furanyl)-7-(2-phenylethyl)-7H-pyrazolo[4,3-e][1,2,4]triazolo[1,5-c]pyrimidin-5-amine, Cat# 2270, 2019), 5-BDBD (5-(3-bromophenyl)-1,3-dihydro-2H-benzofuro[3,2-e]-1,4-diazepin-2-one, Cat# 3579, 2019), and JNJ47965567 (2-(phenylthio)-N-[[tetrahydro-4-(4-phenyl-1-piperazinyl)-2H-pyran-4-yl]methyl-3-pyridinecarboxamide were from Tocris (Bristol, UK). All drugs were used in supra-maximal but selective concentrations based on our previous studies: 20 µM PPADS [23], 100 nM BBG [17], 20 µM 5-BDBD [24], 1 µM JNJ47965567 [25], 100 µM AOPCP [10], and 50 nM SCH58261 [26].

### Model of Presymptomatic PD

**Fig. 1:**
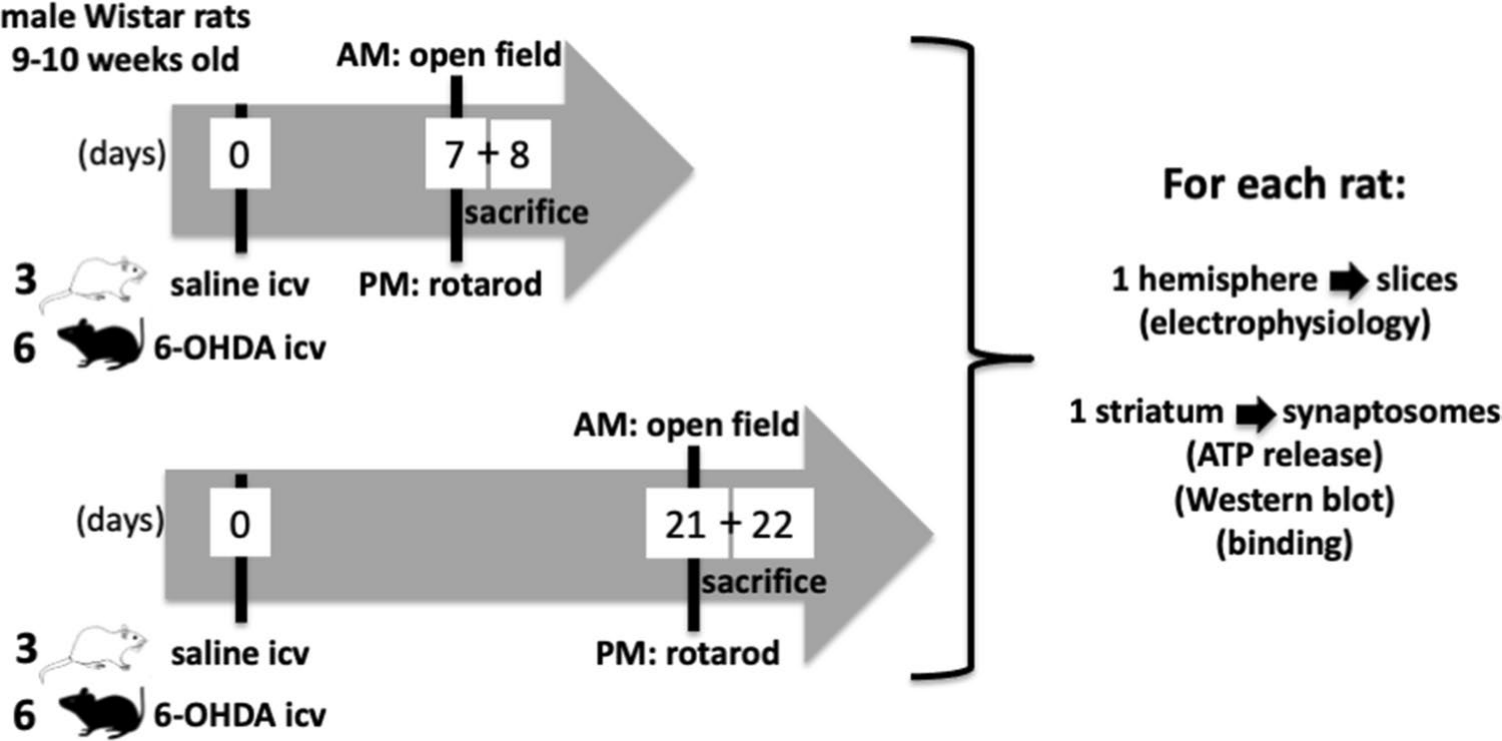
Timeline of the experiments comparing presymptomatic and symptomatic PD rats. The number of animals per group is indicated on the left side of each depicted rats. The number of days is depicted within the arrowed timeline

We used a previously characterized rat model of early PD, based on our previous studies defining the time course of behavioral and neurochemical alterations caused by different doses of 6-hydroxydopamine (6-OHDA, Cat#162,957, 2019, Sigma) applied bilaterally in the dorsolateral striatum, as previously described [21, 22]: as a presymptomatic model of PD model (i.e., with a minor lesion of the nigrostriatal dopaminergic pathway and devoid of measurable motor deficits), we evaluated rats 7 days after the bilateral administration of 20 μg 6-OHDA into the dorsolateral striatum (AP: + 0.2 mm, ML: ± 3.5 mm, DV: − 4.8 mm from bregma and dura), whereas rats were evaluated 21 days after the administration of 20 μg 6-OHDA to model early PD (i.e., with lesions of the nigrostriatal dopaminergic pathways associated with motor deficits). 6-OHDA was delivered in a volume of 3 μL at a rate of 1.0 μL/min, using a Hamilton 10-μL syringe with a 26-gauge needle connected to a 30-gauge cannula. Following injection, the cannula was left in place for 5 min before being retracted, to allow complete diffusion of the drug. Sham-operated rats followed the same protocol except that vehicle (saline) was injected instead of 6-OHDA. The animals were administered intraperitoneally (i.p.) with desipramine (20 mg/kg) (Sigma-Aldrich) 30 min before surgery, in order to protect noradrenergic terminals from 6-OHDA toxicity. The stereotaxic surgeries were performed under ketamine (75 mg/kg)/xylazine (8 mg/kg) i.p. anesthesia, which is optimal for the required time of interventions while affording a complete recovery of all animals. During the post-operative period, animals were maintained heated and hydrated with supplemented hydrated food (apple slices) but were not administered with analgesics which are known to interfere with the adenosine modulation system [27] that is the focus of this study. Six rats per group were used, first tested behaviorally and then sacrificed (half at 3PM, half at 8AM) by decapitation after anesthesia under halothane atmosphere to obtain slices and striatal fractions to carry out electrophysiological and neurochemical studies, as described in the scheme depicted in Fig. 1: although there was only one control group, half the rats from this group were paired with each test group to allow evaluating in parallel each test group and control rats.

### Behavioral Analysis

Behavioral tests were performed between 9AM and 2PM. In the open-field test, rats were allowed to freely explore during 15 min a wooden arena (100 × 100 cm, gray walls and gray floor) to assess their spontaneous locomotor activity by quantifying the total distance travelled, the number of ambulatory episodes, and the number of rearing events [17]. The cylinder test was carried out as previously described [17]. Rats were placed in a transparent cylinder (30 cm high and 20 cm in diameter) to record during a 5-min use of each forelimb during rearing behavior to estimate vertical motor activity. In the accelerated rotarod test, rats were tested for their balance and motor coordination in a single session during which the cylinder rotation speed was progressively increased to measure the latency to fall, as previously described [17].

### Immunohistochemical Quantification of Tyrosine Hydroxylase

Immunohistochemistry was essentially carried out as previously described [17]. Briefly, the animals were anesthetized with sodium thiopental and transcardially perfused with ice-cold phosphate-buffered saline (PBS) followed by 4% paraformaldehyde in PBS. The brains were removed, post-fixed in 4% paraformaldehyde for 16–24 h, and cryoprotected in 30% sucrose for 48 h at 4 °C. The brain was then frozen in dry ice and 50 µm coronal sections were prepared using a cryostat (Leica CM3050S) at − 21 °C. Tyrosine hydroxylase immunodetection was performed in 50 µm free-floating sections, which were washed 3 times for 10 min with PBS and incubated with PBS supplemented with 10% methanol and 1.05% hydrogen peroxide for 40 min at room temperature (RT), to block endogenous peroxidase-like activities. After washing 3 times for 10 min with PBS and blocking endogenous proteins with 10% normal goat serum in PBS supplemented with Triton X-100 (blocking solution) for 2 h at RT, the sections were incubated with the primary antibody (rabbit anti-tyrosine hydroxylase, 1:1000; ref. AB152 from Merck-Millipore) diluted in blocking solution at 4 °C for 48 h. The sections were then washed with PBS before incubation for 2 h at RT with a secondary goat anti-rabbit biotinylated antibody (1:200, Vector Labs) diluted in blocking solution, and washed with PBS. The avidin–biotin-horseradish peroxidase conjugate (ABC Staining System, Santa Cruz Biotechnology) was used for 40 min at RT for amplification of the signal and revealed with DAB Peroxidase Substrate Kit (Vector labs). The reaction was stopped by washing with PBS before mounting on gelatin-coated slides, dried, dehydrated by a gradient of ethanol, and cleared with xylene. Finally, the sections were coverslipped with Entellan (Merck). The stained brain sections were visualized using an Olympus BX41 epi-fluorescent microscope equipped with an Olympus DP71 camera. Immunoreactivity was measured by semi-quantitative densitometric analysis using an image-analysis program (Image J software). The optical densities of striata of the sham-operated group were averaged and the values of other groups calculated as a percentage of that mean.

### HPLC Quantification of Dopamine Levels

Dopamine was quantified by HPLC, essentially as previously described [21]. Samples of the analyzed brain areas were homogenized by ultrasonication in 0.1 M perchloric acid with 0.02% sodium metabisulfite and 10 μM 3,4-dihydroxybenzylamine as internal standard. After centrifugation at 10,000 × *g* for 5 min at 4 °C, the supernatant (20 μL) was separated through a LiChrospher 100 RP-18 (5 μm) cartridge (Merck) fitted into a Manu-cart holder (Merck) with a mobile phase (pH 4.0) consisting of 0.1 M KH_2_PO4, 3 mM octane-1-sulfonic acid sodium salt, 0.1 mM NaEDTA, and 10% (v/v) methanol kept at a flow rate of 1.2 mL/min. The detection was achieved with an Coulochem-II electrochemical detector (ESA, Analytical) with a dual electrode analytical cell (ESA 5011A) set at 250/ − 175 mV for a sensitivity at 0.5 nA/V and the peak areas of the external standards were used to quantify DA levels.

### ATP Release

The release of ATP from purified striatal synaptosomes of rats or mice was measured on-line using the luciferin-luciferase assay [9, 10]. The evoked release of ATP was calculated by integration of the area of the peak upon subtraction of the estimated basal ATP outflow [6, 9, 10]. We always confirmed the integrity of the synaptosomes by quantifying the amount of lactate dehydrogenase and of glutamine in the medium after the assays, as previously described [9, 10].

### Single Nerve Terminal Immunocytochemistry

The immunocytochemical detection of P2XR subunits and P2YR in individual glutamatergic nerve terminals of the striatum was carried out as previously described [28]. Briefly, striatal nerve terminals were platted over poly-L-lysine-coated coverslips, and incubated overnight at 4 °C with the primary guinea pig antibodies against glutamate vesicular transporter type 1 (vGluT1, RRID:AB_2301751) and type 2 (vGluT2, RRID:AB_1587626; both 1:2,000, Chemicon, UK) and either rabbit anti-P2X1 (RRID:AB_2341048; 1:500, from Alomone Labs, Israel), rabbit anti-P2X2 (RRID:AB_2341051; 1:1,000, from Alomone Labs), goat anti-P2X3 (RRID:AB_2158068; 1:300, from Santa Cruz Biotechnology, USA), rabbit anti-P2X4 (RRID:AB_2040058; 1:500, from Alomone Labs), mouse anti-P2X5 (RRID:AB_10847859; 1:100, from Santa Cruz Biotechnology), mouse anti-P2X6 (RRID:AB_2158241; 1:100, from Santa Cruz Biotechnology), rabbit anti-P2X7 (RRID:AB_2040068; 1:10,000, from Alomone Labs), rabbit anti-P2Y1 (RRID:AB_10863775; 1:200, from Abcam), rabbit anti-P2Y2 (RRID:AB_2040078; 1:500, from Alomone Labs), rabbit anti-P2Y4 (RRID:AB_2040080; 1:500, from Alomone Labs), and rabbit anti-P2Y6 (RRID:AB_2040082; 1:300, from Alomone Labs) receptors, all prepared in phosphate-buffered saline with 3% bovine serum albumin. After addition of fluorescent-labelled secondary antibodies (RRID:AB_141954, RRID:AB_2534069, RRID:AB_2556776; 1:200 for all; Invitrogen, Portugal), the preparations were examined under a fluorescence microscope. Images, acquired in each color channel using identical masks, were quantified using the IMAGEJ 1.37v software (RRID:SCR_003070; NIH, Bethesda, MD, USA), to quantify Pearson’s correlation between the different color channels with a significance level > 95% [29, 30].

### Subsynaptic Fractionation

Subsynaptic fractionation of synaptosomes allows an over 90% effective separation of the presynaptic active zone (enriched in SNAP-25), postsynaptic density (enriched in PSD95), and non-active zone fraction or extra-synaptic fraction (enriched in synaptophysin), as previously detailed [28]. Similar amounts of protein from each of these subsynaptic fractions, as well as from the initial synaptosomal preparation, were subject to Western blot analysis [28] to detect each of the P2XR subunits or P2YR using the previously described antibodies against P2X1 (1:500), P2X2 (1:1,000), P2X3 (1:300), P2X4 (1:500), P2X5 (1:100), P2X6 (1:100), P2X7 (1:10,000), P2Y1 (1:200), P2Y2 (1:500), P2Y4 (1:500), and P2Y6 (1:300) receptors, followed by staining with alkaline phosphatase-conjugated secondary antibodies (RRID:AB_2336536; Amersham, UK).

### Density of CD73 and of A_2A_ Receptors

The density of CD73 in synaptic membranes from the striatum was carried out by Western blot analysis, as previously described [10]. Briefly, striatal synaptosomes were solubilized in 5% sodium dodecyl sulfate (SDS; Bio-Rad) supplemented with 2 mM dithiothreitol and 100 mM phenylmethylsulfonyl fluoride and rapidly sonicated. After determining the amount of protein using the bicinchoninic acid method (Pierce), 1/6 volume of 6 × SDS-PAGE sample buffer (8 M urea, 100 mM dithiothreitol, 2% (w/v) sodium dodecyl sulfate, and 375 mM Tris–HCl pH 6.8) was added and electrophoresis was carried out with 20 μg of protein of synaptosomal protein loaded into a 10% SDS-PAGE gel with a 4% concentrating gel under reducing conditions. The proteins were then transferred to polyvinylidene difluoride membranes (GE Healthcare), that were blocked for 1 h at RT with 5% low-fat milk in Tris-buffered saline medium pH 7.6, containing 0.1% Tween 20 (TBS-T). The membranes were then incubated overnight at 4 °C with the rabbit polyclonal anti-CD73 primary antibody (1:300, Santa Cruz). After three washing periods of 15 min with TBS-T, the membranes were incubated for 2 h at RT with an alkaline phosphatase-tagged secondary antibody (Invitrogen) diluted in TBS-T containing low-fat milk. After three 15-min washes with TBS-T, the membranes were incubated with Enhanced Chemi-Fluorescence kit (GE Healthcare) and visualized in a VersaDoc 3000 imaging system with the assistance of Quantity One software (Bio-Rad).

The density of A_2A_R in synaptosomes was estimated by radioligand binding assays using a supra-maximal concentration of ^3^H-SCH58261 (2 nM; offered by E.Ongini, Schering-Plough, Italy), as previously described [31]. The binding reactions were carried out at RT (22–23 °C) with 0.1–0.2 mg of protein for 1 h in 50 mM Tris and 10 mM MgCl_2_ with 4 U/mL of adenosine deaminase (Sigma). Specific binding was determined by subtraction of non-specific binding, measured using 3 μM XAC (Tocris).

### Electrophysiology

Electrophysiological recordings were carried out at 32 °C as previously described [18, 22] in coronal slices containing the dorsolateral striatum (400 µm thick) by extracellularly recording population spike (PS) responses upon positioning the recording electrode in the dorsolateral striatum and the bipolar concentric stimulation electrode in the white matter above the dorsolateral striatum, delivering stimuli (250–350 µA) of 0.1 ms duration at a frequency of 0.05 Hz. An input/output curve was first carried out to choose an intensity of stimulation yielding 40–50% of the maximum response. Long-term potentiation (LTP) was induced as optimized [18, 22], by 3 trains of 100 Hz pulses (1 s duration, 1 every 10 s). LTP was quantified as the percentage of change of the average amplitude of the five potentials taken between 55 and 60 min after LTP induction in relation to the average PS amplitude measured during the 10 min that preceded LTP induction.

### Statistics

The sample size for each experimental set was defined based on our previous experience using the animal models [21, 22] or each analytical procedure (e.g., [9, 10]). Data are mean ± SEM of *n* experiments (*n* = number of animals) and significance was considered at *p* < 0.05 using either a paired *t* test to assess effects of any individual drug or treatment (comparing pre- and post-treatment values), a two-tail Student’s *t* test with Welsh correction for comparison between two groups, and one-way ANOVA (followed by Bonferroni’s post hoc test) or two-way ANOVA (followed by a Newman-Keuls post hoc test) for comparison of multiple groups, after assessing normality using a Shapiro–Wilk test. Identification and consequent removal of outliers was made by the Grubb’s test. Statistical analyses were carried out using the GraphPad Prism 8.1.1 software (San Diego, CA, USA, RRID:SCR_002798). No randomization was performed to allocate animals to the different experimental groups and no exclusion criteria were used, resulting in the inclusion of all animals. We did not carry out a power analysis, since we relied on our previous experience of the models used and drugs tested to pre-define the sample size.

## Results

### ATP Release from Striatal Nerve Terminals

**Fig. 2:**
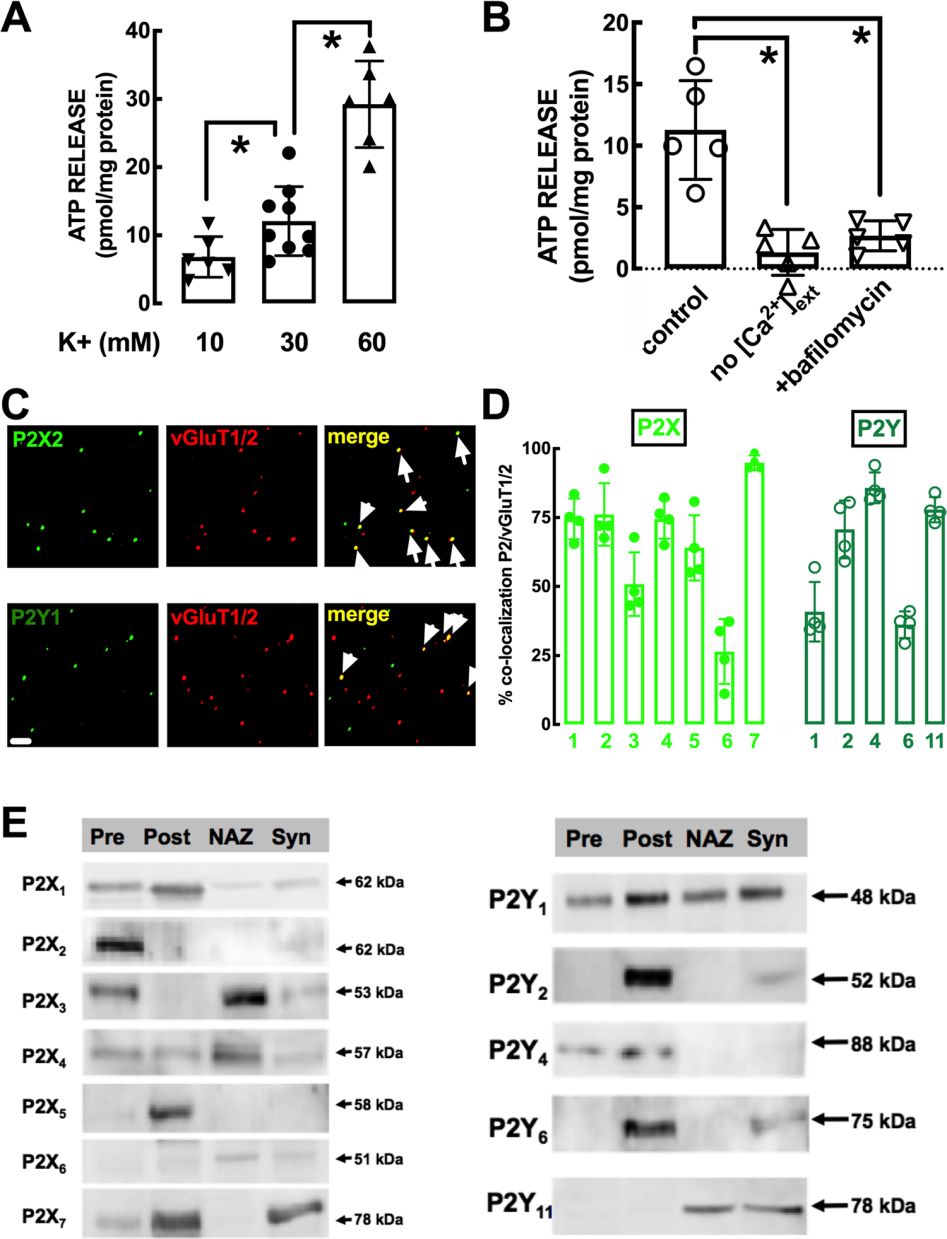
Intensity-dependent release of vesicular ATP and localization of different P2X and P2Y receptors in glutamatergic nerve terminals and within synapses in the mouse striatum. **A** The evoked release of ATP, evaluated with a luciferin-luciferase enzymatic assay, was triggered by exposure of mouse striatal synaptosomes to different concentrations of K^+^ (isomolar substitution of Na^+^ by K^+^ in the medium) and was larger at more intense stimulation. **B** ATP release was abolished in the absence of added extracellular calcium and inhibited in the presence of the vesicular proton pump inhibitor bafilomycin (100 nM), indicating a vesicular evoked release of ATP from nerve terminals. Data in **A** and **B** are mean ± S.E.M of 6–9 experiments (number of different animals tested); *p < 0.05 using a one-way ANOVA followed by Bonferroni’s post hoc test. **C** Representative photographs of immunocytochemistry staining of striatal nerve terminals with P2X2 receptor (upper row) and P2Y1 receptor subunit (lower row) and their co-localization with markers of glutamatergic nerve terminals (vesicular glutamate transporter type 1 and 2 – vGluT1/2) as highlighted by the arrows in the merged image. The scale bar is 10 μm. **D** Histograms representing the average co-localization of the different P2X receptor (P2XR) subunits or different P2YR in glutamatergic nerve terminals (i.e., labeled with vGluT1/2) from the mouse striatum. Data are mean ± S.E.M of 3 mice. **E** Subsynaptic localization in striatal synapses of the different P2XR subunits or different P2YR, assessed by Western blot analysis in the initial striatal synaptosomal preparation (Syn) and in purified extracts of the presynaptic active zone (Pre), the postsynaptic density (Post), and the extra-synaptic regions or non-active zone (NAZ). The blots are representative from two similar subsynaptic fractionations from the mouse striatum

We used synaptosomes to isolate the specific contribution of the presynaptic component for ATP release, which can be triggered with a chemically induced depolarization, using increasing extracellular K^+^ in the range of 5–60 mM [32, 33]. Accordingly, K^+^ (10–60 mM) triggered a concentration-dependent enhancement of ATP release from mouse striatal synaptosomes (Fig. 2A). Notably, whereas the release of classical neurotransmitters such as glutamate or GABA from synaptosomes reaches a near maximal value at 30 mM K^+^ [32, 33], the evoked ATP release was lower when using lower extracellular K^+^ concentrations (6.82 ± 1.22 and 12.08 ± 1.69 pmol/mg protein, *n* = 6–9, at 10–30 mM K^+^) and was larger when triggered with a higher K^+^ extracellular concentration (60 mM: 29.24 ± 2.59 pmol/mg protein, *n* = 6) (Fig. 2A). The 30 mM K^+^-evoked ATP release was abrogated in the absence of extracellular calcium (1.33 ± 0.83 pmol/mg protein, *n* = 5; *t* = 1.601, *p* = 0.185 vs. 0) and decreased by 75.09 ± 4.94% (*n* = 5; *t* = 4.577; *p* = 0.007 vs. control) in the presence of the vesicular proton pump inhibitor bafilomycin (100 nM), indicating a vesicular ATP release from striatal nerve terminals (Fig. 2B).

### Synaptic and Subsynaptic Localization of P2R in the Striatum

We next used a double immunocytochemistry staining of individual striatal nerve terminals to identify if P2X and P2Y receptors were located in striatal glutamatergic synapses, by quantifying the co-localization of markers of glutamatergic synapses (vesicular glutamate transporters type 1 and type 2, vGluT1/2) and different P2XR and P2YR [28]. Figure 2C displays two examples of photographs of an immunocytochemical staining of striatal nerve terminals with P2X2 and P2Y1 receptor subunit and their co-localization with markers of glutamatergic nerve terminals. The average data are presented in Fig. 2D, showing that most P2XR subunits and P2YR were present in glutamatergic terminals of the striatum. Furthermore, a subsynaptic fractionation of striatal synapses identified a qualitative presynaptic enrichment of P2X2 and a qualitative postsynaptic enrichment of P2X1, P2X5, P2X7, P2Y1, P2Y2, P2Y4, and P2Y6 (Fig. 2E). This prompts considering P2R as likely targets of synaptically released ATP to control corticostriatal transmission.

### Role of P2R in the Control of Corticostriatal Transmission

**Fig. 3:**
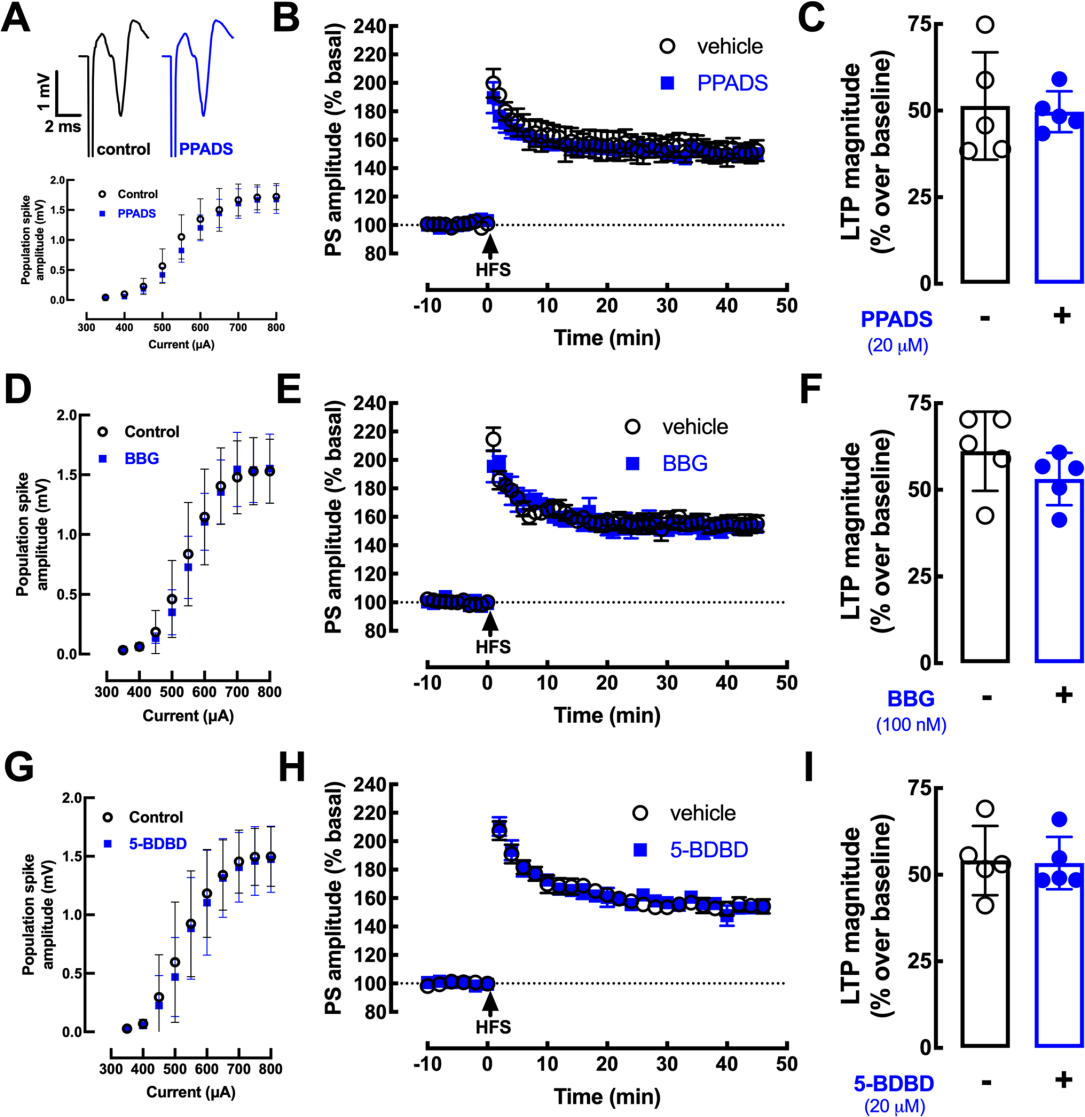
P2 receptor antagonists do not affect corticostriatal transmission or plasticity. **A**, **D**, **G** Neither population spikes (PS) recorded in the dorsal striatum upon cortical stimulation in mouse brain slices nor input/output curves nor **B**, **C**, **E**, **F**, **H**, **I** high-frequency stimulation (HFS)–induced long-term-potentiation (LTP) were modified in presence of: **A**–**C** the generic P2R antagonist pyridoxalphosphate-6-azophenyl-2′,4′-disulfonic acid (PPADS, 20 μM); **D**–**F** the preferring P2X7 receptor (P2X7R) antagonist Brilliant Blue-G (BBG, 100 nM); **G**–**I** the selective P2X4R antagonist 5-(3-bromophenyl)-1,3-dihydro-2Hbenzofuro[3,2-e]-1,4-diazepin-2-one (5-BDBD, 20 μM). Data are mean ± SEM of 5 experiments (number of different animals tested). No significant alterations of either basal synaptic transmission or LTP magnitude were observed with any of the tested drugs (Student’s *t* test at *p* < 0.05)

To test if P2R are involved in the ability of synaptically released ATP to affect corticostriatal transmission, we tested the effects of some P2R antagonists. The generic P2R antagonist PPADS (20 µM), which we previously showed to modify hippocampal transmission [23], did not affect basal corticostriatal synaptic transmission (Fig. 3A), yielding super-imposable input/output curves (Fig. 3A), and was also devoid of effects on high-frequency-induced long-term potentiation (LTP) at corticostriatal synapses (Fig. 3B, C). Since PPADS has a low efficiency to antagonize P2X4R and P2X7R, we tested more selective antagonists of both these receptors. The P2X7R antagonist BBG (100 nM) was devoid of effects on basal transmission and LTP at corticostriatal synapses (Fig. 3D–F), the same occurring when testing another more selective P2X7R antagonist JNJ47965567 (1 µM, *n* = 2; data not shown). The P2X4R antagonist 5-BDBD (20 µM), which we previously showed to modify hippocampal transmission [24], was also devoid of effects on basal transmission and LTP at corticostriatal synapses (Fig. 3G–I). Altogether these findings indicate that P2R are not involved in the control of corticostriatal transmission by synaptically released ATP.

### Role of ATP-Derived Adenosine in the Control of Corticostriatal Transmission

**Fig. 4:**
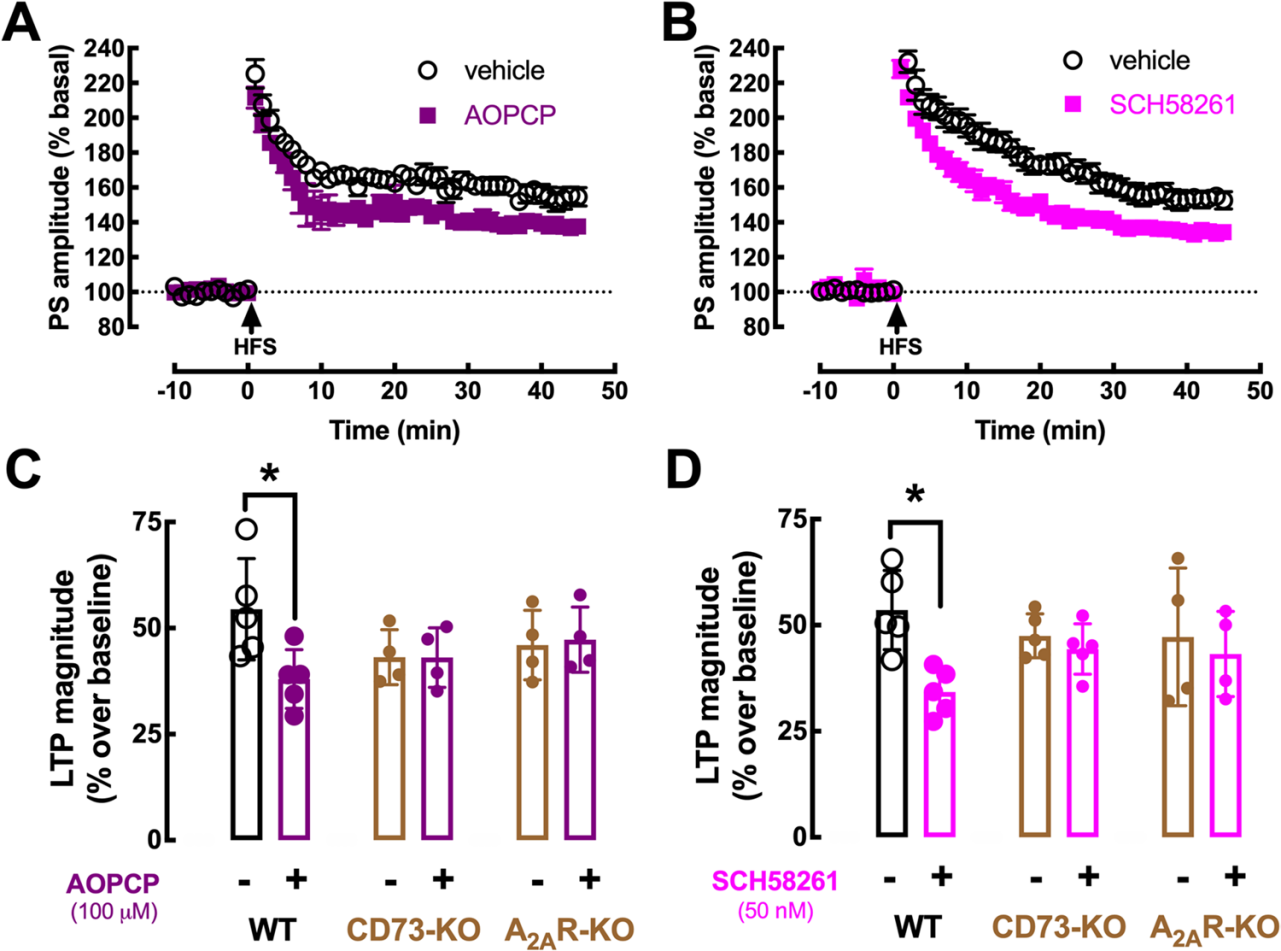
ATP-derived extracellular adenosine formed by CD73 activates A_2A_R to control corticostriatal LTP. The high-frequency stimulation (HFS)–induced enhancement of the amplitude of population spikes (PS) – long-term potentiation (LTP) – recorded in corticostriatal synapses from wild-type (WT) mice slices was decreased in the presence of the CD73 inhibitor α,β-methylene ADP (AOPCP, 100 μM) (**A**), or of the selective A_2A_R antagonist SCH58261 (50 nM) (**B**). **C** The effect of AOPCP was abrogated in slices from either CD73 knockout (KO) mice or A_2A_R-KO mice. **D** Likewise, the effect of SCH58261 was also abrogated in slices from either CD73-KO or A_2A_R-KO mice. This shows that CD73-derived extracellular adenosine selectively activates A_2A_R to control corticostriatal LTP. Data are mean ± SEM of 4–6 experiments (number of different animals tested); *p < 0.05 using a Student’s *t* test with Welsh correction for comparison between two groups

To test if synaptically released ATP could modify corticostriatal transmission indirectly through adenosine receptors upon its extracellular catabolism, we first tested the impact of the CD73 inhibitor AOPCP at a supra-maximal and selective concentration to inhibit the extracellular generation of ATP-derived adenosine involving CD73 activity in striatal nerve terminals [9]. AOPCP (100 µM) did not affect basal excitatory corticostriatal synaptic transmission (5.01 ± 2.06% modification of population spike amplitude; *n* = 5; *p* = 0.062 with a paired Student’s *t* test comparing population spike amplitude before and after AOPCP administration), excluding an association of ATP-derived adenosine with tonic A_1_R activation at corticostriatal synapses (see also [34]). In contrast, AOPCP decreased LTP amplitude (from 54.45 ± 5.35% over baseline without AOPCP to 37.99 ± 6.18% over baseline with AOPCP; *n* = 5, *p* < 0.05, unpaired *t*-test) in slices from wild-type (WT) mice (Fig. 4A, C). In slices from CD73-KO mice, corticostriatal LTP magnitude was lower than in WT mice (42.74 ± 3.20%; *n* = 10, *p* < 0.05 vs. WT, unpaired *t*-test) and AOPCP was devoid of effects (Fig. 4C).

Since adenosine modulation of synaptic plasticity is mostly operated by A_2A_R (reviewed in [13]), we attempted to confirm if the effect of AOPCP was due to a selective prevention of the CD73-mediated formation of extracellular adenosine tonically activating A_2A_R. Indeed, the A_2A_R antagonist SCH58261 (50 nM) phenocopied the effect of AOPCP on corticostriatal LTP (53.57 ± 4.18% without, 34.25 ± 2.47% with SCH58261, *n* = 5, *p* < 0.05, unpaired *t*-test; Fig. 4B, D), without affecting basal synaptic transmission (3.31 ± 3.22% modification of population spike amplitude; *n* = 5, *p* = 0.123 vs. 0%). Most importantly, the effect of AOPCP on LTP was not observed in A_2A_R-KO mice (Fig. 4C), which also displayed a LTP magnitude that tended to be lower than in WT mice (46.63 ± 4.21%; *n* = 8, *p* = 0.174 vs. WT, unpaired *t*-test). Accordingly, the impact of SCH58261 on LTP amplitude was lost in striatal slices from A_2A_R-KO mice (Fig. 4D) as well as from CD73-KO mice (Fig. 4D) indicating that ATP-derived adenosine by CD73 is critically involved in the control by A_2A_R of corticostriatal LTP.

### Role of ATP-Derived Adenosine in Controlling Corticostriatal Transmission in a Rat Model of PD

A_2A_R antagonists are now approved as a new therapeutic tool to manage PD [16] and we recently defined that targeting CD73 was equieffective to A_2A_R antagonists to alleviate motor symptoms in an animal model of PD [9]. We now tested if the overfunction of this CD73-mediated A_2A_R activation pathway is involved in the onset of motor symptoms in PD, as occurs in convulsions-induced neurodegeneration [30]. This entails that CD73-mediated A_2A_R overfunction should be observed in the presymptomatic phase of PD.

**Fig. 5:**
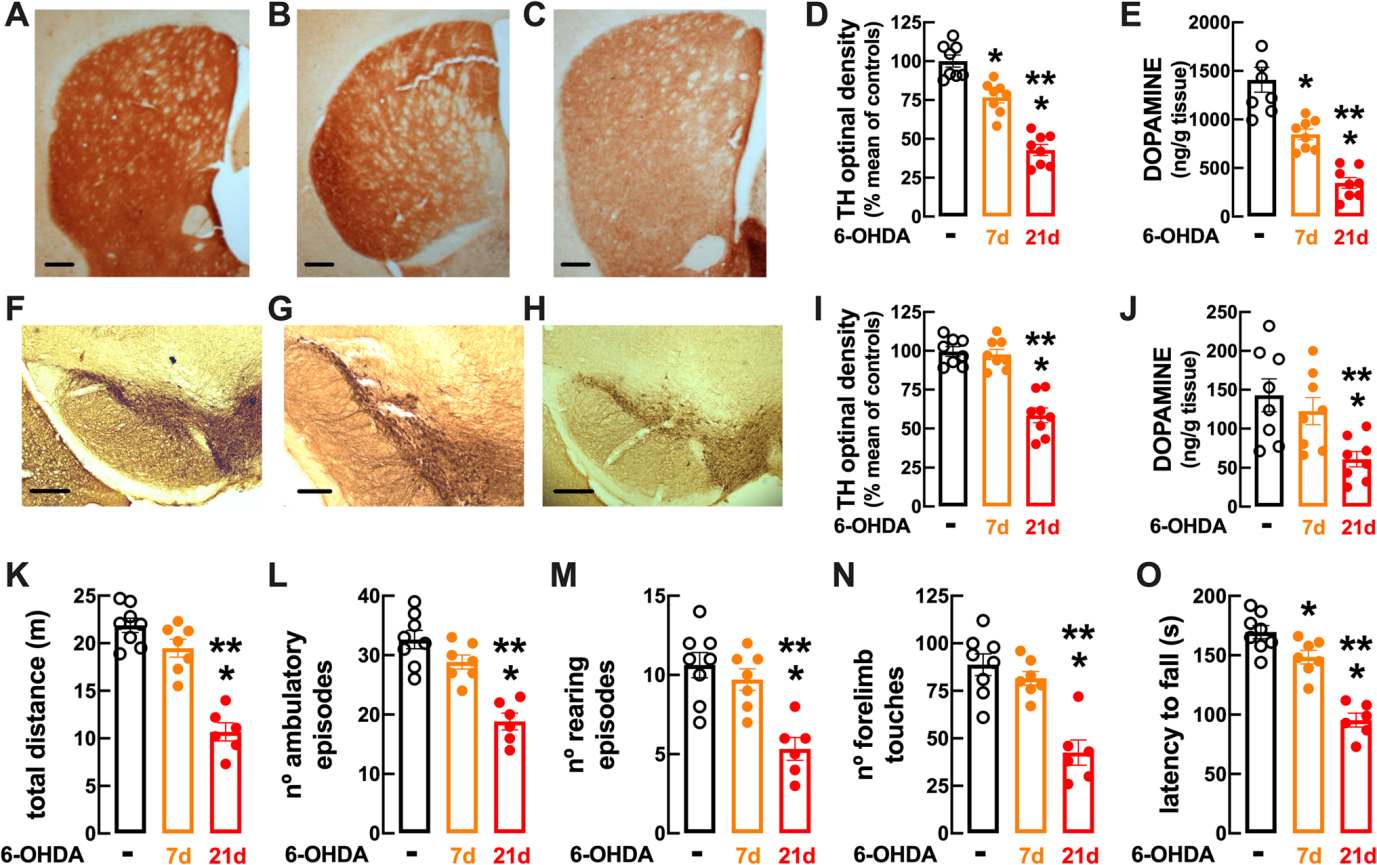
Characterization of a rat model of the presymptomatic phase of Parkinson’s disease (PD). In a rat model of PD onset based on the bilateral administration of 6-hydroxydopamine (6-OHDA, 20 μg in each striatum), it is possible to distinguish a presymptomatic phase at 7 days after 6-OHDA exposure and a symptomatic phase at 21 days after exposure. Representative coronal sections of the dorsolateral striatum (**A**–**C**; away from the injection site; scale bars: 200 μm) or substantia nigra (**F**–**H**; scale bars: 150 μm) stained for the dopaminergic marker, tyrosine hydroxylase (TH), from saline-treated rats and 7 days (**B**, **G**) or 21 days (**C**, **H**) after the bilateral administration of 6-OHDA (20 μg in each striatum), with the presentation of the immunohistochemical quantification of TH density in the striatum (**D**) or substantia nigra (**I**). The average dopamine levels in the striatum (**E**) and in the mesencephalon (**J**) confirmed a partial depletion of dopamine in the striatum with no alteration in the mesencephalon at 7 days after 6-OHDA administration, which was aggravated in the striatum and became evident in the mesencephalon at 21 days after 6-OHDA administration. This translated into a non-significant modification of motor performance at 7 days, which evolved into an overt motor dysfunction at 21 days, as heralded by the total distance travelled (**K**), the number of ambulatory episodes (**L**), the number of rearing events in an open-field test (**M**), as well as the number of touches with both paws in the cylinder test (**N**) and the time before falling in the rotarod test (**O**). Data are mean ± SEM of 6–8 rats per group; *p < 0.05 vs. control and **p < 0.05 vs. 7 days, using either a one-way ANOVA followed by Bonferroni’s post hoc test

To test this hypothesis, we took advantage of our recently characterized titration of different doses of 6-OHDA to trigger presymptomatic and motor modifications pertinent to PD in rats [21, 22]. Thus, as depicted in Fig. 5, the bilateral intra-striatal administration of 20 μg 6-OHDA caused a progressive depletion of tyrosine hydroxylase density in the striatum (Fig. 5A–D), which was 76.78 ± 3.61% of control after 7 days (*F*_2,14_ = 6.31; *p* < 0.05 vs. controls) and 42.89 ± 3.57% of control after 21 days (*F*_2,14_ = 15.50; *p* < 0.05 vs. controls and *F*_2,14_ = 9.19; *p* < 0.05 vs. 7 days), and a parallel decrease of the dopamine levels in the striatum, which were lower (*F*_2,14_ = 5.76; *p* > 0.002) at 21 days (346 ± 55.3 ng/mg tissue, *n* = 8) than after 7 days (847 ± 57.8 ng/mg tissue, *n* = 8) compared to control (1409 ± 129 ng/mg tissue, *n* = 8) (Fig. 5E). In the substantia nigra, the decrease of tyrosine hydroxylase density was not observed at 7 days (*p* = 0.935 vs. control) but became evident at 21 days (*F*_2,14_ = 10.77; *p* < 0.05 vs. control) (Fig. 5F–I) and, similarly, the dopamine levels in the mesencephalon were still not altered at 7 days (*p* = 0.671 vs. control) but were lower at 21 days (*F*_2,14_ = 4.87; *p* = 0.007 vs. control) (Fig. 5J). In accordance with this discrete dopaminergic depletion limited to the striatum at 7 days after 6-OHDA administration, these rats did not yet display evident motor dysfunction, namely a non-significant decrease of spontaneous locomotion assessed as the total distance travelled in an open field (*p* = 0.140; Fig. 5K), a non-significant decrease of the number of ambulatory episodes in an open field (*p* = 0.156; Fig. 5L), a non-significant decrease of the number of rearing episodes in an open field (*p* = 0.654; Fig. 5M), a non-significant decrease of vertical activity assessed as the number of touches with both paws in the cylinder test (p = 0.608; Fig. 5N), and a decrease of motor coordination performance in the rotarod test (*F*_2,12_ = 3.80, *p* = 0.038; Fig. 5O). In accordance with the more extensive dopaminergic dysfunction in the striatum and in the substantia nigra, motor dysfunction became evident 21 days after the bilateral administration of 6-OHDA (20 μg) as illustrated by a significant decrease in the open field of the total distance travelled (*F*_2,12_ = 12.71, *p* < 0.05 vs. controls; Fig. 5K), of the number of ambulatory episodes in an open field (*F*_2,12_ = 9.62, *p* < 0.05 vs. controls; Fig. 5L) and of the number of rearing episodes (*F*_2,12_ = 7.00, *p* < 0.05 vs. controls; Fig. 5M); furthermore, there was also a non-significant decrease of the number of touches with both paws in the cylinder test (*F*_2,12_ = 8.43, *p* < 0.05 vs. controls; Fig. 5N), and a decrease of motor coordination in the rotarod test (*F*_2,12_ = 12.82, *p* < 0.05 vs. controls; Fig. 5O) at 21 days after the bilateral administration of 6-OHDA (20 μg). Overall, these data are indicative of a PD-like status at 21 days after the bilateral administration of 6-OHDA (20 μg), whereas at 7 days the insidious dopaminergic dysfunction still without evident motor alteration is suggestive of a presymptomatic stage of PD.

**Fig. 6:**
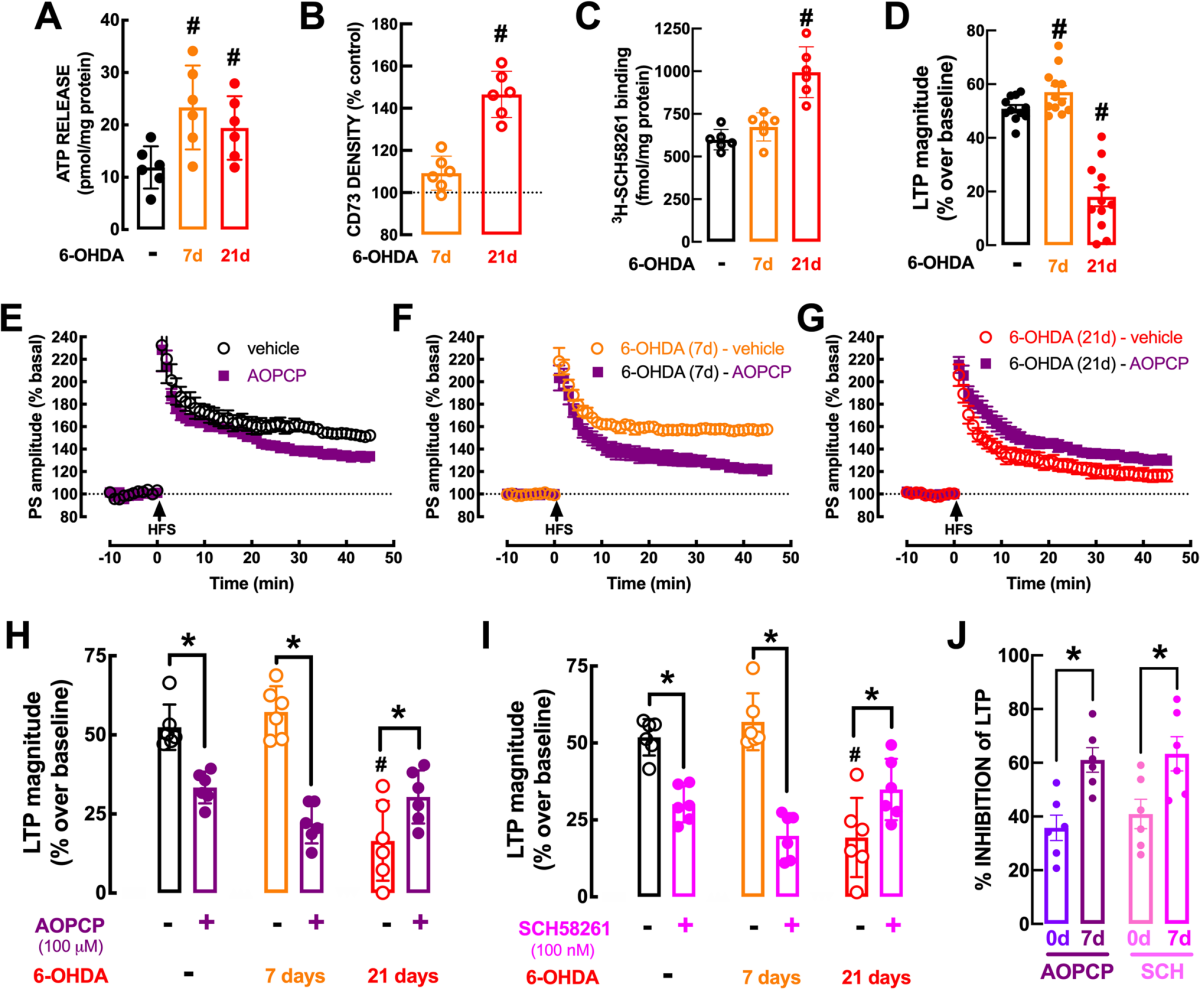
Increased ATP release and contribution of CD73-mediated formation of extracellular adenosine activating A_2A_R to bolster corticostriatal synaptic plasticity in the presymptomatic phase of Parkinson’s disease (PD). **A** The 30 mM K^+^-evoked ATP release from rat striatal nerve terminals is increased during the presymptomatic phase of PD at 7 days after administration of 6-hydroxydopamine (6-OHDA, 20 μg in each striatum) and remains upregulated throughout the protocol. **B**, **C** However, the protein levels of CD73 assessed by Western blot (**B**), and A_2A_R density measured as the binding of the A_2A_ receptor antagonist ^3^H-SCH58261 (2 nM) (**C**), were not modified in striatal synapses in the presymptomatic phase and were only increased with the onset of motor symptoms at 21 days after 6-OHDA challenge. **D** In the presymptomatic phase of PD, corticostriatal long-term potentiation (LTP) displayed a larger amplitude, which decreased upon onset of the motor symptoms. **E** The recording of population spikes (PS) in rat slices showed that the CD73 inhibitor AOPCP (100 μM) decreased the high-frequency stimulation (HFS)–induced corticostriatal LTP and this effect was larger during the presymptomatic phase of PD (**F**) and shifted into a normalization of the depressed LTP during the symptomatic phase (**G**). Average alteration of the amplitude of corticostriatal LTP at different times after 6-OHDA administration and similar qualitatively effect of either blocking CD73 with AOPCP (100 μM) (**H**) or blocking A_2A_R with SCH58261 (50 nM) (**I**), both of which had a larger effect on corticostriatal LTP during the presymptomatic phase of PD (**J**). Data are mean ± SEM of 6 rats per group; #*p* < 0.05 vs. control in the absence of drugs (black bars or dashed line) using a one-way ANOVA followed by Bonferroni’s post hoc test (**A**–**D**) and *p < 0.05 between indicated bars using a two-way ANOVA followed by a Newman-Keuls post hoc test (**G**–**I**)

As reported for mice (Fig. 2A), ATP was also released upon chemical depolarization of rat striatal synaptosomes with K^+^ (Fig. 6A). Notably, the 30 mM K^+^-evoked release of ATP from striatal nerve terminals was larger in the presymptomatic period of our progressive PD model (7 days after 6-OHDA: 23.34 ± 3.27 pmol/mg protein vs. 11.89 ± 1.65 pmol/mg protein in controls; *F*_2,10_ = 4.482, *p* < 0.05) and is maintained elevated into the symptomatic period (after 21 days: 19.41 ± 2.48 pmol/mg protein, *n* = 6) (Fig. 6A). However, this augmented release of ATP was not accompanied by an increased protein amount of CD73 (*p* = 0.106 vs. control) or of A_2A_R density (*p* = 0.444 vs. control) in striatal synapses during the presymptomatic period at 7 days after 6-OHDA challenge, which was only present during the symptomatic period, at 21 days after 6-OHDA challenge (CD73: *F*_2,15_ = 59.10, *p* < 0.001 vs. control; A_2A_R: *F*_2,15_ = 24.35, *p* < 0.001 vs. control) (Fig. 6B, C).

We then tested if this increased synaptic ATP release in the presymptomatic phase would drive a bolstered CD73-mediated A_2A_R modulation of corticostriatal synaptic plasticity in presymptomatic PD, as occurs in early AD [10] or shortly after convulsions [30]. Importantly, the magnitude of corticostriatal LTP was larger during the presymptomatic phase of PD (Fig. 6D). In parallel, whereas in slices from vehicle-treated rats AOPCP decreased LTP magnitude by 37.09 ± 3.07% (*n* = 6; Fig. 6E, H), the AOPCP-induced inhibition of corticostriatal LTP magnitude was increased (*F*_1,20_ = 9.81, *p* < 0.05) to 62.67 ± 8.11% (*n* = 6; Fig. 6F, H, J) in slices collected from rats 7 days after 6-OHDA administration. Directly blocking A_2A_R with SCH58261 phenocopied this increased impact of ATP-derived adenosine on LTP magnitude during the presymptomatic PD period (Fig. 6I), as heralded by the larger inhibition of corticostriatal LTP magnitude caused by 50 nM SCH58261 (*F*_1,20_ = 8.302, *p* < 0.05) in slices from rats 7 days after 6-OHDA exposure (65.59 ± 10.87% inhibition, *n* = 6) compared to vehicle-injected rats (40.71 ± 3.77% inhibition, *n* = 6) (Fig. 6I, J). These findings indicate an increased role of CD73-mediated A_2A_R overfunction in the presymptomatic phase of PD, which is ensured by an increased availability of ATP-derived adenosine without requiring an upregulation of CD73 or A_2A_R in striatal synapses. In other words, it is the overactivity of this pathway, driven by an increased ATP release, rather than the alteration of the density of its constituents that ensures the overactivation of A_2A_R, concluded based on the larger amplitude of the effects of AOPCP or SCH58261 on LTP magnitude. Importantly, this A_2A_R overactivation driven by an increased ATP release contributes to an increased LTP magnitude during the presymptomatic phase of PD (58.78 ± 2.53% over baseline) compared to control conditions (50.84 ± 1.38% over baseline; *t*_19_ = 2.82, *p* = 0.011), when considering the pooled data from the two experimental groups displayed in Fig. 6H and I (Fig. 6J).

As observed in other brain areas afflicted in rodents modeling symptomatic brain diseases [9, 30], there is a shift in the role of A_2A_R between still normal and clearly abnormally functioning brain circuits that is associated with an upregulation of synaptic A_2A_R. Thus, at 21 days after 6-OHDA administration, motor symptoms are evident (Fig. 5K–O) and corticostriatal LTP amplitude is decreased (20.54 ± 5.25% over baseline, *F*_1,20_ = 11.65, *p* < 0.05 vs. vehicle-treated); the blockade of A_2A_R now partially recovered (rather than inhibited as in control conditions) corticostriatal synaptic plasticity (LTP amplitude: 34.88 ± 4.07% over baseline in the presence of 50 nM SCH58261, *n* = 6; *p* < 0.05 vs. the absence of SCH58261) (Fig. 6I). This shift of function of A_2A_R in the control of corticostriatal LTP between 7 and 21 days after 6-OHDA administration, probably results from the observed upregulation of A_2A_R in striatal synapses observed at 21 days after 6-OHDA administration (see Fig. 6C). The same qualitative shift was observed between presymptomatic (7 days) and symptomatic (21 days) upon blockade of CD73, re-enforcing the tight association between CD73 and A_2A_R: thus 21 days after 6-OHDA administration, AOPCP now partially recovered corticostriatal LTP amplitude (18.34 ± 5.13% over baseline in the absence and 30.38 ± 3.43% over baseline in the presence of 100 μM AOPCP, *n* = 6, *p* < 0.05) (Fig. 6G, H).

## Discussion

The present study prompts two main intertwined conclusions: first, the use of synaptosomes allowed defining the pattern of ATP release from stimulated striatal nerve terminals and the distribution of P2 receptors in striatal synapses, which were surprisingly devoid of major modulatory effects on corticostriatal plasticity; instead, the use of different knockout mice allowed defining the importance of the ATP release-CD73-A_2A_R activation pathway to control LTP at corticostriatal synapses. The subsequent use of a tentative rat model of presymptomatic PD then allowed concluding that this pathway is overactivated during the presymptomatic phase of an animal model of PD. In fact, during presymptomatic PD, there was an increased A_2A_R-mediated modulation of corticostriatal LTP driven by an increased synaptic release of ATP without alteration of the density of CD73 and of A_2A_R. In contrast, upon symptomatic PD, there was an increased density of CD73 and of A_2A_R now contributing to a reduced corticostriatal LTP magnitude driven by ATP-derived adenosine.

The presently reported evoked release of ATP from striatal nerve terminals confirms previous reports of the production of extracellular ATP as a signaling molecule in the striatum, namely by nerve terminals [9, 35]. We now observed that the evoked release of ATP from nerve terminals seems to follow a pattern different from that of classical neurotransmitters since moderate to intense depolarization (10 to 30 mM K^+^) somewhat increased ATP release, whereas it causes near maximal release of classical neurotransmitters, and ATP release was near doubled from intense (30 mM K^+^) to near supra-maximal depolarization (with 60 mM K^+^) whereas the release of classical neurotransmitters is nearly saturated under these conditions [32, 33]. This re-enforces previous observations supporting that the presynaptic release of ATP follows a pattern different from that of classical neurotransmitters [5, 6, 36] being released in disproportionally larger amounts at larger intensities of nerve recruitment; this is in agreement with the reported storage of ATP in vesicles different from these storing classical neurotransmitters [36, 37]. This hints at a particular role of ATP in conditions associated with more intense synaptic activity, which are associated with the engagement of synaptic plasticity processes.

Surprisingly, endogenous ATP is devoid of direct effects on corticostriatal LTP, as gauged by the lack of effects of the generic P2R antagonist PPADS, in contrast to other brain regions [38, 39]. Moreover, a selective P2X4R antagonist, 5-BDBD, was also devoid of effects in spite of the involvement of P2X4R in the control of LTP in the hippocampus [40, 41]. As previously reported [34, 42], corticostriatal transmission was also unaffected by the tested P2R antagonist. Thus, although most P2XR and P2YR were identified in striatal synapses and in particular in glutamatergic synapses (see Fig. 2), we cannot yet ascribe any function to these synaptic P2R in the control of glutamatergic synaptic transmission or plasticity in the striatum. However, P2R control several striatal-mediated behavioral [43, 44] and neurochemical responses [45] as well as striatal damage [17, 46] probably through non-synaptic mechanisms.

The main finding of the present study is the identification of the mechanisms operated by synaptically released ATP to control synaptic plasticity in the striatum: this involves the local extracellular catabolism of ATP into adenosine to activate A_2A_R that are engaged in the modulation of corticostriatal LTP. This extends to the striatum the existence of an ATP-CD73-A_2A_R pathway dedicated to the control of synaptic plasticity, as was previously reported to occur in the control of hippocampal synaptic plasticity [10, 26]. It also confirms the previously described association of CD73 and A_2A_R in the control of striatal-dependent behavioral responses [8] and in the control of behavioral and neurochemical alteration in PD models [9]. A major new finding was the observation that this released ATP-CD73-A_2A_R pathway controlling corticostriatal LTP is overfunctioning in the presymptomatic phase of PD, i.e., 7 days after the administration of 6-OHDA when mood modifications are already present but motor dysfunction is still not significant [21, 22]. Glutatamergic corticostriatal as well as thalamocortical synapses is affected since early PD [47–50] with increased firing rates and bursting activity of the corticostriatal pathway being observed in the presymptomatic phase of PD [51, 52]. Accordingly, we now observed that corticostriatal LTP magnitude was larger in the presymptomatic phase of PD. This suggests that abnormal glutamatergic transmission in the striatum may contribute to the pathophysiology of PD (reviewed in [20]) and A_2A_R-mediated potentiation of glutamatergic function may be a contributing factor (reviewed in [13]). Indeed, in other conditions of brain dysfunction such as early Alzheimer’s disease [9]) and convulsions-induced neurodegeneration [30], we have also reported an early upregulation of the pathway released ATP-CD73 density and activity-A_2A_R density and function controlling the early abnormally increased plasticity of hippocampal LTP that pre-dated the onset of synaptotoxicity and memory symptoms [9, 30]. Thus, the observation of an increased magnitude of corticostriatal LTP and increased participation of A_2A_R to corticostriatal LTP magnitude is also suggestive of a role of released ATP-CD73-A_2A_R overfunction in the etiology of PD. This is in accordance with the previously proposed role of synaptic A_2A_R in the adaptive processes of glutamatergic excitotoxicity aggravating striatal dopamine loss and the emergence of motor symptoms in established PD (reviewed in [53]). Three aspects need to be highlighted: first, it is the overactivity of this pathway rather than the alteration of the density of its constituents that ensures the overactivation of A_2A_R. In other words, the increased contribution of A_2A_R to the increased corticostriatal LTP magnitude during presymptomatic PD is not due to an increased density of CD73 or of A_2A_R but rather to an increased activity of CD73 and of A_2A_R driven by an increased synaptic release of ATP, i.e., it is a change of software rather than of hardware. Second, this A_2A_R overfunction in the presymptomatic phase of PD is not expected to be the sole or main determinant defining the magnitude of LTP; in fact, LTP magnitude is dependent on a large variety of executors and A_2A_R are only modulators fine-tuning different key elements responsible for the implementation of synaptic changes, such as, for instance NMDA receptors (e.g., [26]). Our data show that A_2A_R activation has a greater participation to bolster LTP magnitude during presymptomatic PD, but this conclusion should not be linked to alterations of the absolute magnitude of corticostriatal LTP, which optimal point may well be re-adjusted to cope with the initial alterations in the afflicted circuit, through mechanisms independent of A_2A_R. Thirdly, it is important to stress that our sole focus was to characterize the role of endogenously produced adenosine on corticostriatal plasticity; this implies the use of receptor antagonists and we carefully avoided testing A_2A_R agonists such as CGS21680, which could recruit populations of striatal A_2A_R that are not engaged by endogenous adenosine under our experimental conditions and could even enroll A_1_R, as occurs in amygdala synapses [54].

Whereas the role of A_2A_R in corticostriatal synapses of normal and presymptomatic PD animals is a facilitation of LTP, it is transformed into an opposite effect in the symptomatic phase of PD, as previously observed in different brain areas after different brain insults [29, 30, 55]. Although the mechanisms underlying this shift of the impact of A_2A_R await to be clarified, they are probably related with the upregulation of A_2A_R upon noxious brain condition, the pleiotropic ability of A_2A_R to engage numerous transducing systems (reviewed in [13]), and the shift of the transducing system of A_2A_R under excitotoxic conditions [56]. Thus, as observed in other models of neurodegenerative disorders such as Alzheimer’s disease [9] and convulsions-induced neuronal damage [30], the role of A_2A_R is initially designed to facilitate synaptic plasticity in non-diseased conditions; however, A_2A_R overfunction causes an abnormal increase of synaptic plasticity that seems to contribute to the onset of synaptic damage. This synaptotoxic process seems to be closely associated with an upregulation of A_2A_R and the activation of these new A_2A_R now dampens synaptic plasticity. Thus, A_2A_R antagonists seem to have a dual role in controlling these aberrant synaptic changes, controlling the onset of synaptic dysfunction and the established synaptic dysfunction; however, these two beneficial effects seem to be based on opposite effects of A_2A_R exerted by a different number of A_2A_R operating in novel conditions of decreased synaptic plasticity resulting from a lower efficiency of the machinery involved in the implementation of these plastic changes, such as inotropic NMDA and AMPA receptors, voltage sensitive calcium channels, protein kinases, and phosphatases or metabolic supporting systems, just to name a few (reviewed in [57]).

In conclusion, the present study defined that synaptically released ATP is converted by CD73 into adenosine to activate A_2A_R controlling synaptic plasticity at corticostriatal glutamatergic synapses. Moreover, this released ATP-CD73-A_2A_R pathway is overfunctioning in the presymptomatic phase of PD, bolstering plasticity at striatal glutamatergic synapses, which is hypothesized to contribute to the onset of motor symptoms in PD. The translation of these findings to humans tentatively prompt extending the use of the recently FDA-US-approved A_2A_R antagonist [16] from only co-adjuvants of motor control in Parkinsonian patients to neuroprotective drugs delaying the onset of motor symptoms. However, such translation to humans needs to be considered with care in view of the limitations of the animal model of presymptomatic PD that we now exploited; in fact, whereas the administration of 6-OHDA is useful to mimic a progressive dopaminergic dysfunction in the rodent brain in areas similar to these afflicted in PD patients and leads to motor impairments in rodents that are reminiscent of PD features, this model fails to trigger a modification α-synuclein processing and associated neurotoxicity that are of central importance in the pathogenesis of human PD (for a review see [58]).

## Data Availability

The data supporting the findings of this study are available from the corresponding author upon reasonable request.

## Abbreviations

A_1_R: Adenosine A_1_ receptor
A_2A_R: Adenosine A_2A_ receptor
AOPCP: α,β-Methylene ADP
BBG: Brilliant Blue-G
5-BDBD: 5-(3-Bromophenyl)-1,3-dihydro-2H-benzofuro[3,2-e]-1,4-diazepin-2-one
KO: Knockout
LTP: Long-term potentiation
NAZ: Non-active zone
6-OHDA: 6-Hydroxydopamine
P2R: P2 receptors
PBS: Phosphate-buffered saline
PD: Parkinson’s disease
Post: Postsynaptic density
PPADS: Pyridoxalphosphate-6-azophenyl-2′,4′-disulfonic acid
Pre: Presynaptic active zone
PS: Population spike
RT: Room temperature
PSD95: Postsynaptic density protein of 95 kDa
SCH58261: 2-(2-Furanyl)-7-(2-phenylethyl)-7H-pyrazolo[4,3-e][1,2,4]triazolo[1,5-c]pyrimidin-5-amine
SNAP-25: Synaptosomal-associated protein of 25 kDa
Syn: Synaptosomal preparation
TBS-T: Tris-buffered saline medium containing 0.1% Tween 20
TH: Tyrosine hydroxylase
vGluT1/2: Vesicular glutamate transporters type 1 and type 2
